# A mouse model of lamellar intrastromal femtosecond laser keratotomy: ultra-structural, inflammatory, and wound healing responses

**Published:** 2011-11-17

**Authors:** R.I. Angunawela, R. Poh, S.S. Chaurasia, D.T. Tan, J.S. Mehta

**Affiliations:** 1 Tissue Engineering and Stem Cell Group, Singapore Eye Research Institute, Singapore; 2 Singapore National Eye Centre, Singapore; 3 Department of Ophthalmology, Yong Loo Lin School of Medicine, National University of Singapore, Singapore; 4 Department of Clinical Sciences, Duke-NUS Graduate Medical School, Singapore

## Abstract

**Purpose:**

The availability of knockout mouse species provide a highly versatile platform for critically examining the corneal wound healing response. We aimed to develop and characterize the wound healing response in a mouse model of intrastromal femtosecond laser (FSL) keratotomy.

**Methods:**

An intrastromal lamellar dissection using a Visumax FSL was performed on 16 wild type mice (C57BL6) . The energy level was optimized at 150nJ. The FSL was programmed to perform a lamellar dissection at 50 µM depth without sidecut. The flap was not lifted. Fellow eyes were used as controls. Slit lamp photography and confocal microscopy were performed immediately before the mice were sacrificed 4 h, 1, 3, and 7 days post surgery. Corneas were harvested for immunocytochemistry, transmission electron microscopy (TEM) and light microscopy (LM).

**Results:**

Confocal microscopy showed an absence of keratocytes in the area immediately surrounding the dissection plane. The dissection plane and individual FSL plasma cavitation bubbles were clearly evident on TEM. There was evidence of Keratocyte cell death along the laser resection plane on TEM. LM revealed the dissection plane at a 20 µM depth, although not all epithelial cell layers were intact. Staining for monocytes using antibodies for CD11b (cluster of differentiation 11b) showed early migration at the peripheries at 4 h that increased at 24 h and became more central in treated corneas (p<0.001). Apoptotic cells were evident on TUNEL (terminal deoxynucleotidyl transferase dUTP nick end labeling) assay in the immediate ablation zone and were significantly raised at 4 and 24 h (p<0.001). Ki67 (Kiel 67 protein) positive proliferating keratocytes are evident at 3 days and increased significantly by 7 days (p<0.001). Minimal fibroblast (cluster of differentiation 90, CD90) transformation was seen at 1 week. No myofibroblasts were detected.

**Discussion:**

We have demonstrated that FSL lamellar cuts can be effectively performed on mice and that this model exhibits typical signs of the corneal wound healing response. This model could provide a ubiquitous platform in which to study corneal wound healing responses in both wild type and knockout mice species. The ability to create such a lamellar pocket may be utilizzd for intrastromal drug delivery.

## Introduction

Elucidation of the mouse genome, together with the availability of transgenic mouse species and a wealth of disease models, makes the humble laboratory mouse an unsurpassed model for understanding and investigating human disease. In the context of corneal disease, the availability of knockout mice species offers a unique platform from which to critically probe, and better understand the genetic and molecular basis of corneal wound healing events, immune responses, and pharmacogenetics [1].

Many mutant mice species with altered corneal and anterior segment anatomy have been characterized and described in the literature [2]. The mouse mutant ACa 23 (small nucleolar RNA, H/ACA box 23) was recently found to have a significantly reduced corneal thickness and enlarged anterior chamber and could possibly serve as a model for keratectasia after refractive surgery [3]. Absence of the scavenger receptor CD36 (cluster of differentiation 36) in the corneal epithelium of CD36 knockout mice results in spontaneous bacterial keratitis and provides a useful insight into the antimicrobial defense mechanisms of the cornea [4]. These examples represent just a few of the multitude of potential disease models currently available to researchers.

Previous investigators have used murine models to evaluate mechanisms of corneal transplant rejection, allergic eye disease and wound healing responses [5-9]. These models can be challenging when they require surgery that is analogous to a human operation be performed on the mouse eye [10]. Mouse corneal wound healing models, particularly in the context of refractive surgery, have not been extensively used due to the relative resistance of the mouse cornea in forming haze after excimer laser treatment [11]. However, more recently, Mohan et al. [11] have described a technique of irregular phototherapeutic keratectomy that effectively induced corneal haze and provides a model for studying wound healing and myofibroblast biology, albeit in the setting of corneal epithelial injury.

The femtosecond laser (FSL) has recently emerged as a 21st century alternative to the surgeon’s scalpel. It has now superseded the microkeratome as the instrument of choice for flap creation for laser in situ keratomileusis (LASIK), and is able to perform previously difficult, complex and precise multiplanar incisions for keratoplasty [12,13]. The FSL laser is a near infrared laser that creates a corneal resection plane by photodisruption and plasma formation. Coalescence of these plasma cavitation bubbles results in resection plane, and when combined with the precise spatial optics of the laser, provides an incredibly accurate method of incising the cornea. Currently available FSLs require coupling of the cornea with the laser, using a docking interface that is designed for the dimensions of the human cornea.

In this study we aimed to investigate the possibility of using the FSL as a tool for consistently creating an intra-stromal lamellar incision or pocket within the mouse cornea, and have developed and optimised a technique for achieving this with the standard FSL docking interface. We have also characterized the ultrastructural and wound healing responses of the FSL laser in this pure model of stromal injury in the mouse.

## Methods

### Animals

The Institutional Animal Care and Use Committee of SingHealth approved all animal experiments described in this work. All animals were treated according to tenets of the Association for Research in Vision and Ophthalmology’s statement for the Use of Animals in Ophthalmic and Vision Research.

C57BL/6 mice were bred and maintained at the SingHealth Experimental Medical Centre (Singapore General Hospital, Singapore). The mice were anesthetized by intraperitoneal injection of a 0.1 ml ketamine/xylazine mixture containing 2 mg/ml xylazine hydrochloride (Troy Laboratories, Smithfield, Australia) and 20 mg/ml ketamine hydrochloride (Ketamine, Parnell Laboratories, Alexandria, Australia) before the surgery was performed as described below. Mice were euthanized at various time points after surgery (4, 24, 72 h and 1 week) by intraperitoneal overdose anesthesia with sodium pentobarbitol.

All mice had pre and post-operative slit lamp photography with the Nikon FS-3V zoom photo slit lamp (Nikon, Tokyo, Japan). Post operatively 4 mice had confocal corneal microscopy performed 4 h after surgery with the Heidelberg retina tomography HRT3 (Heidelberg Engineering GmbH, Heidelberg, Germany). A carbomer gel (Vidisic; Mann Pharma, Berlin, Germany) was used as immersion fluid. All corneas were examined centrally with at least 3 *z*-axis scans through from epithelium to endothelium. In vivo confocal micrographs were analyzed with the Heidelberg Eye Explorer version 1.5.1 software (Heidelberg Engineering GmbH).

### Femtosecond laser procedure

All surgery was performed with a 500 kHz femtosecond laser (VisuMax, Carl Zeiss Meditec, Jena, Germany). The laser parameters were as follows: 50 μm flap thickness; 3 mm flap diameter; 150 nJ power; and spot distance and tracking spacing of 4.8 μm/4.8 μm for lamellar dissection.

The Visumax laser has several docking interface treatment pack sizes, ranging from small to large that establish limbal or peripheral corneal suction once docked with the human eye. The machine must detect an established vacuum pressure before the laser firing sequence can be initiated. This acts as a safeguard to ensure globe fixation during surgery. We used a large size treatment pack for the purpose of this experiment since it has the flattest radius of curvature. We were able to ‘dock’ the treatment pack to the mouse eye by prolapsing each globe using gentle finger pressure while simultaneously lifting the mouse into contact with the treatment cone. This was done while directly observing the mouse’s eye through the laser’s operating microscope, until maximal contact with the cone was achieved. To simulate effective vacuum pressure, the vacuum tubing was first kinked and obstructed before activating the vacuum. The machine thus detected an effective vacuum pressure and allowed the laser firing sequence to be initiated. The laser was stopped after the lamellar firing sequence and before side cuts were made. The epithelium was hence not breached during the procedure. The lamellar incision was left undisturbed in all cases, and each mouse was returned to its enclosure to recover from anesthesia.

In an initial pilot experiment to evaluate best laser parameters, we treated both eyes of 3 mice with energy settings ranging from 50 to 150 nJ. These three mice were sacrificed and enucleated 4 h later. Whole globes were fixed for transmission electron microscopy and light microscopy of the FSL resection plane. Based on these pilot experiments, we found that a 150 nJ laser energy setting resulted in a consistent and predictable resection plane. This setting was used in all subsequent experiments.

In total 16 eyes of 16 mice underwent FSL treatment as described above. Fellow eyes were used as controls. Slit lamp photography was performed pre and post-operatively and 4 mice underwent confocal microscopy as described at the 4 h time point. Four mice were sacrificed and enucleated at each of the 4, 24, 72 h, and 1 week time points after surgery. Tissue was preserved for immunohistochemistry.

### Transmission electron microscopy (TEM)

Specimens were fixed in 2% glutaraldehyde and 2% paraformaldehyde in 0.1M sodium cocodylate buffer, pH 7.4 (Electron Microscopy Sciences, Washington, PA) overnight at 4 °C. They were secondarily fixed in 1% osmium tetroxide tetroxide and potassium ferrocynaide (Electron Microscopy Sciences). After extensive rinsing with sodium cacodylate buffer, they were dehydrated in a graded series of ethanol, and embedded in Araldite (Electron Microscopy Sciences). All semi-thin sections of 0.5–1 µm thickness were cut with a Reichert-Jung Ultracut E Ultramicrotome (C. Reichert Optische Werke AG, Vienna, Austria), counter-stained with toluidine blue/basic fuchsin stain and examined using an Axioplan, Zeiss Light Microscope (Carl Zeiss, Jena, Germany). All ultra-thin sections of 60–80 nm were collected on copper grids, doubled-stained with uranyl acetate and lead citrate for 20 min each, then viewed and photographed on a Philips EM 208S Transmission Electron Microscope (FEI Electron Optics BV, Eindoven, The Netherlands) at 100kV.

### Tissue fixation and sectioning

For immunofluorescent staining, whole globes were embedded in optimal cutting temperature (OCT) embedding compound (Leica Microsystems, Nussloch, Germany). Frozen tissue blocks were stored at −80 °C until sectioning. Serial sagittal corneal 8μm sections were cut using a Microm HM550 cryostat (Microm, Walldorf, Germany). Sections were placed on polylysine-coated glass slides and air dried for 15 min and processed for immunohistochemistry.

### Immunohistochemistry

Sections were fixed with 4% paraformaldehyde (Sigma, St. Louis, MO) for 20 min, washed with 1× PBS, blocked with 4% BSA (Sigma) in 1× PBS, 0.15% Triton X-100 (Sigma) for 1 h, and incubated with either rat monoclonal antibody against CD11b (BD PharMingen) diluted 1:100 in the blocking solution, α-smooth muscle actin (α-SMA, Dako Cytomation, Glostrup, Denmark) diluted 1:50 or with 1:100 rat monoclonal antibody against K_i_-67 (Invitrogen, Carlsbad, CA) at 4 °C overnight. After washing with 1× PBS, the sections were incubated with goat anti-rat Alexa Fluor 488-conjugated secondary antibody (Invitrogen, Carlsbad, CA) at room temperature for 1 h. Slides were then mounted with UltraCruz Mounting Medium containing DAPI (Santa Cruz Biotechnology, Santa Cruz, CA). For negative controls, non-immune serum was used in place of the specific primary antibody. Sections were observed and imaged with a Zeiss Axioplan 2 fluorescence microscope (Zeiss, Oberkochen, Germany). Normal corneas were used as uninjured controls.

### TUNEL assay

To detect apoptotic cells, a fluorescence-based TUNEL (terminal deoxynucleotidyl transferase dUTP nick end labeling) assay (In Situ Cell Death Detection Kit, Roche Applied Science, Indianapolis, IN) was used according to the manufacturer’s instructions.

### Quantification of positively staining cells

Four corneas from each time point were used to quantify positively staining cells for CD90 (cluster of differentiation 90), Ki67 (Kiel 67 protein), CD18/Thy1 (cluster of differentiation 18/thymocyte differentiation antigen 1), α-SMA (alpha smooth muscle actin), and TUNEL in the tissues by a previously described technique [11]. Briefly, positively staining cells in randomly selected, non-overlapping, full-thickness central and peripheral corneal columns extending from the anterior stromal surface to the posterior stromal surface were counted using several sections from each cornea at each time point. Six central and 2–3 peripheral columns on each side (4–6 total) were counted in each case. The diameter of each column was a 400× microscope field.

### Statistical analysis

Data were expressed as mean±standard deviation (SD) where appropriate. The p value was determined using ANOVA with the Graphpad Prism statistical package (GraphPad Software, Inc. La Jolla, CA). Data were considered to be statistically significant when p<0.05.

## Results

### Slit lamp photography

**Figure 1:**
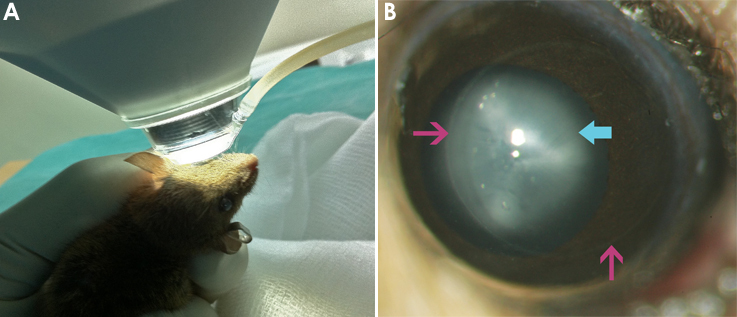
Surgical positioning and post-operative images of mouse eye. **A**: Photo showing positioning of the mouse cornea against the treatment cone. The mouse eye was partially prolapsed and brought into contact with the cone. The area of surface contact was visualized directly through the operating microscope. **B**: Color slit lamp image showing the mouse cornea 4 h after femtosecond laser (FSL) keratotomy . The circumferential outline of the FSL incision is indicated by the arrow. The cornea is clear (note the clearly visible iris details) but with some corneal edema present at this time point. A cataract is present behind the cornea and appears opaque (cyan arrow).

The area of laser ablation was evident 4 h after FSL as a circumferentially demarcated area within the cornea. The corneas of all mice were generally clear, although in some there were localized areas of tissue edema within the treatment zone (Figure 1). The corneas of mice at other time points were clear and without evidence of opacity.

### TEM ultra-structural analysis

**Figure 2:**
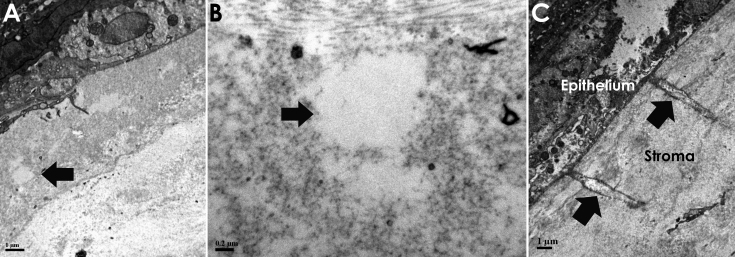
Transmission electron micrographs (TEM) of the stroma within the area of the femtosecond laser keratotomy. An individual cavitation is highlighted (**A**, arrow). In the same image, epithelium is seen superiorly (dark area containing cell contents). **B**: An individual cavitation in greater detail. **C**: Streaks consistent with laser pulses within the stroma, note that the separation between streaks is roughly 5 microns.

TEM scans of the mouse cornea after FSL keratotomy showed a horizontal intra-stromal plane of cavitations at a regular depth from the corneal epithelium. Each of these localized cavitations measured in the region of 1 µm (Figure 2A,B) without evidence of adjacent collagen disruption. Adjacent keratocytes were structurally intact. We also observed evidence of intrastromal streaks (Figure 2C) which corresponded to the position of the laser pulses.

### Confocal microscopy

**Figure 3:**
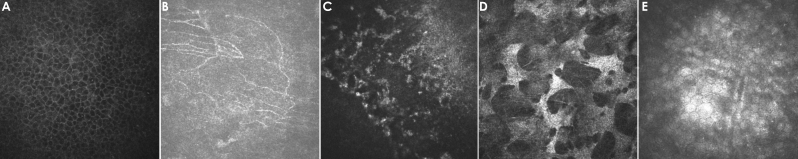
Confocal images through the mouse cornea 4 h after femtosecond laser keratotomy (FSL). **A**: Healthy normal epithelial layer. **B**: Normal distribution of sub-basal nerve fibers. **C**: Fragmentation and debris of cellular matter in the region of the FSL injury. **D**: Normal posterior corneal keratocytes. **E**: Healthy hexagonal endothelial cells. All images were taken with the Heidelberg HRT3 with the Rostock corneal module.

Confocal microscopy was performed 4 h after FSL keratotomy (Figure 3). An intact epithelial morphology was detected, as was a normal sub-epithelial distribution of nerve fibers. In the plane of the FSL incision there was clear evidence of cellular fragmentation, debris and more reflective elements, perhaps representing extracellular aggregations or apoptotic keratocytes. Posterior to this plane we observed a normal network of keratocytes, and further back, the normal morphology of an intact endothelial cell layer. Injury was thus limited to the immediate plane of the FSL incision.

### Immunohistochemistry

#### TUNEL assay

**Figure 4:**
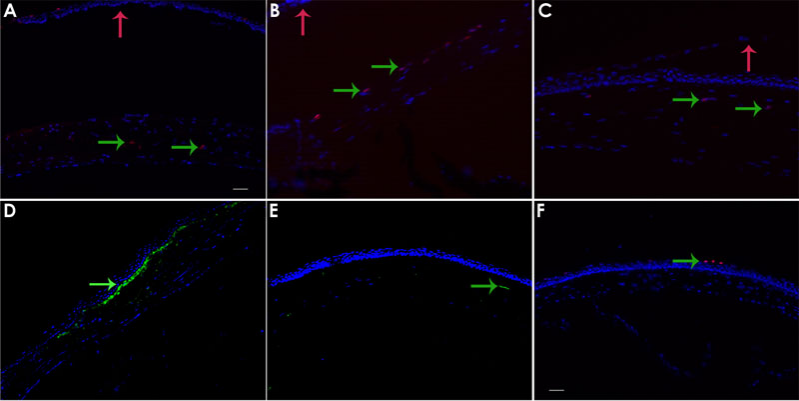
Immunohistochemistry of mouse corneas at various time points. Representative images of immuno-positive staining (green arrows indicate positively staining cells) for (**A**) apoptotic/TUNEL positive cells at 4 h, central cornea. **B**: CD11b positive inflammatory cells, peripheral cornea at 4 h and (**C**) CD11b in the central cornea at 4 h. **D**: Ki67 positive proliferating cells at 1 week and (**E**) shows CD90 positive fibroblastic cells at 1 week. **E**: TUNEL staining in a wild type uninjured mouse cornea, 3 positively staining epithelial cells are indicated. Cell nuclei were stained with DAPI. These image time points were chosen as they represented maximal staining for the particular marker. Epithelial separation occurred in some samples during cryosection and the displaced epithelium is indicated by the red arrows to aid orientation. Scale bar is 50 µm.

**Figure 5:**
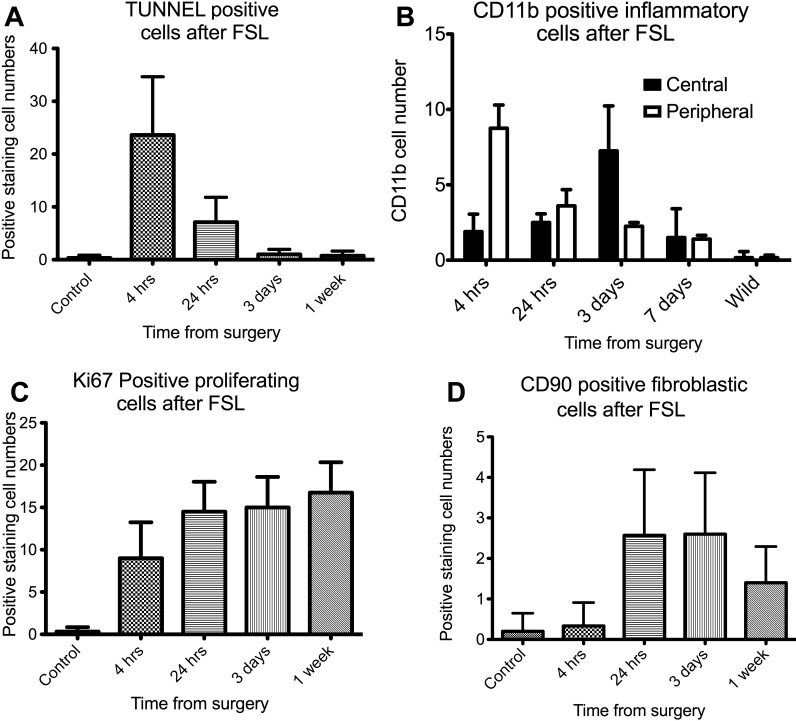
Quantification of positive immunohistochemical staining. Graphs showing quantification of immuno-positive cells for (**A**) apoptosis (TUNEL), (**B**) inflammatory cells (CD11b) showing differential staining in central (black columns) and areas peripheral to the laser dissection (white columns), (**C**) proliferating cells (Ki67) and (**D**) fibroblastic cells (CD90) over 4, 24, 72 h, and 1 week after FSL (femtosecond laser) keratotomy. Error bars indicate standard deviation.

Maximal cellular apoptosis was detected at 4 h and was also significantly raised at 24 h in this model (p<0.001). TUNEL positive cells were detected at other time points, but the numbers of cells were not significant (Figure 4A and Figure 5A).

#### Inflammatory cell response: CD11b positive staining

Inflammatory cells staining positively for CD11b were significantly elevated in the peripheral cornea in the mouse at 4 h after FSL (p<0.001), while the central cornea had expressed few inflammatory cells at this time point. By 3 days however, the central cornea had a significantly elevated number of cells compared to the periphery (p=0.001). This suggests that inflammatory cells first appear in the peripheral region of the cornea and then migrate more centrally to the zone of injury within a few days. Inflammatory cell numbers had decreased from their peak by the 7th post-operative day (Figure 4B and Figure 5B).

#### Cellular proliferation after surgery: Ki67 positive cells

Proliferating keratocytes were detected at 4 h after injury and subsequently increased at 24 h, 3 days (p=0.0187) and 7 day (p=0.002) time points (Figure 4C and Figure 5C). These cells were localized to the area adjacent to the plane of the FSL incision. Maximal proliferating cell numbers were detected at 7 days after surgery.

#### Fibroblast and myofibroblast cells: CD90/Thy 1 and α-SMA positive cells

A small increase in cells displaying a fibroblastic phenotype on immunochemistry was detected from 24 h onwards (p=0.042). These cell numbers did not increase significantly over the week of follow up (Figure 4D and Figure 5D).

We did not detect any positive staining for α-SMA at any time point in treated or control eyes indicating an absence of myofibroblasts during the time period of the study (images not shown). Antibody functionality was validated against a positive control.

## Discussion

The laboratory mouse is an unsurpassed animal model for studying human diseases due to the abundant availability of mutant species, and the close homology of the human and mouse genomes [14,15]. The physical dimensions of the mouse eye and resulting difficulty in performing manual surgery using conventional instrumentation however complicates accurate ocular surgical models in this animal. In this study we have demonstrated that it is possible to adapt and use a conventional clinical femtosecond laser platform to perform intrastromal lamellar surgery on the mouse eye.

The FSL laser causes tissue photo-disruption, which results in the formation of plasma that rapidly expands and collapses, to create an interlamellar (collagen lamellae) cavitation at a precise depth [16]. In the TEM analyses of these cavitations in the mouse cornea we were able to demonstrate distinct localized plasma cavitations with dimensions of ~1 µm. The size of these plasma cavitations is in part related to the energy settings of the laser (we found that a minimal energy of 150 nj resulted in a consistent resection plane). The coalescence of adjacent cavitations determined by the inter-spot spacing (spot and line separation) results in a potential plane of separation that can be dissected manually by the surgeon. In the clinical situation this allows the dissection of LASIK flaps, arcuate keratotomies, and various shapes for corneal keratoplasty.

The mouse cornea has a thickness of 100–130 µm, which is 4–5 times thinner than a normal human cornea. The relative thinness of the mouse cornea makes predictable and repeatable manual lamellar dissection highly challenging. We aimed to produce a lamellar resection plane at a 50 µm depth, although light microscopic analysis of corneal sections after FSL in this study showed a depth of ~30 µm. Some superficial corneal epithelial layers were absent in these sections, possibly as a result of histological preservation, and probably partly account for the disparity between intended and measured depth of the incision plane. The need to prolapse the mouse globe through gentle pressure and manually hold the animal up to the interface cone must also result in some inadvertent movement during the firing sequence (which takes less than 10 s), and could lead to a depth inaccuracy, as would the pressure with which the mouse was applied to the interface cone and the resulting area of contact. The Visumax laser is capable of creating multiplanar horizontal curved incisions that facilitate the excision of an intra-stromal lenticule. This capability has been exploited clinically for the refractive correction of myopia through extraction of a precisely shaped refractive lenticule from the anterior stroma (Refractive Lenticule EXtraction, ReLEx™; Carl Zeiss Meditec) without the need for conventional excimer laser ablation of the cornea, and hence our study is pertinent with respect to the evaluation of the corneal response to isolated femtosecond laser exposure. The intrastromal lamellar pocket that was created by the FSL may also be used as a pocket for drug delivery or tissue transplantation.

Haze is the clinical manifestation of the corneal wound healing response that results in abnormal extracellular matrix deposition and differentiation of passive corneal keratocytes into reflective myofibroblasts [17,18]. Typically, this requires epithelial injury and disruption of the epithelial basement membrane, which allows numerous cytokines and growth factors to establish an epithelial-stromal axis of cellular communication that modulates the stromal wound healing response [19-23]. There was no evidence of loss of transparency in any of the treated corneas during the period of this study, although this would be expected, especially as there was no physical flap lift performed. Previous studies have demonstrated that myofibroblast differentiation occurs rarely after epithelial injury in mice, and that when it does do so, tends to occur 4–6 weeks after injury [12]. We did not identify any myofibroblasts in this study and this is consistent with the purely stromal nature of this injury model. An intra-stromal inflammatory response occurs during the first 3 days after FSL treatment in this model as evidenced by CD11b monocyte detection, initially in the peripheral cornea and then presumably after migration, in the central cornea. Early FSLs were known to cause a delayed inflammatory response leading to localized neuritis that manifested in patients as transient light sensitivity syndrome [24]. This phenomenon has largely disappeared with the reduced energy delivered by the new generations of faster FSLs. While some cells are immediately vaporized by laser photodisruption, other cells in close proximity undergo a slow involutional form of cell death known as apoptosis that can be detected by TUNEL assay. In this study apoptotic cells were largely limited to the stroma surrounding the immediate lamellar dissection plane of the FSL, and were maximally detected between 4 to 24 h after injury. Individual cellular injury leading to apoptosis may occur in the context of the FSL as a consequence of localized thermal, pressure and shockwave sequelae [25]. Ki67, a marker of cellular proliferation was detected maximally in this model after 24 h, and continued to increase at 1 week, indicating the attempted repopulation of the stroma by the remaining stromal keratocytes. Fibroblastic cells indicated by CD90/Thy1 staining were detected in small numbers at a week after the initial FSL surgery. The inflammatory and wound healing responses observed in this model are similar to CD11b positive monocyte invasion and apoptotic responses reported by other researchers following epithelial scrape injuries in mice [26,27].

Corneal stromal cellular responses vary dependent on whether the stromal injury occurs together with an epithelial injury (as in photorefractive kertectomy, PRK), or without an epithelial injury (as in LASIK). The former results in a more robust wound healing response, thought to be due to disturbance of the epithelial basement membrane, which allows access of cytokines and growth factors into the stroma and modulates their subsequent response. In this experiment we simply performed a lamellar FSL dissection, leaving the epithelium undisturbed. An analogous procedure would have been a simple microkeratome pass through the cornea as in LASIK, with the flap left undisturbed, although this would, result in a rim of fibrotic tissue around the flap [28]. This FSL model is thus a relatively pure model of stromal injury, as the epithelial basement membrane remains entirely intact, as long as the flap is left unlifted. In keeping with this we did not detect any myofibroblast differentiation of resident cells during the time period of the study. Anecdotally, FSL flaps in LASIK, are more firmly adherent compared with microkeratome flaps, probably as a consequence of a FSL induced wound healing response [29]. Thermal, pressure, and shockwave effects on the stromal tissue are likely to be markedly different between the mechanisms of cutting with the FSL and microkeratome, thereby resulting in differential wound healing responses in the stroma.

In conclusion we have demonstrated that the FLS laser, with some adaptation, is a useful and precise instrument for performing intrastromal corneal lamellar surgery in the mouse, resulting in a cascade of cellular wound healing sequelae that a typical of stromal injury without epithelial disturbance. This method represents a relatively pure form of stromal only injury, and together with the availability of transgenic mouse species, is a useful paradigm for further investigation of stromal biology and the effect of specific genes on these responses. The ability to create a precise and predictable stromal pocket may also be used a route of drug delivery into the cornea.
